# Effect of different loads on facet joint motion during lumbar lateral bending in sitting position

**DOI:** 10.1186/s13018-024-04533-1

**Published:** 2024-01-13

**Authors:** Ye Han, Wei Yuan, Shaosong Sun, Bao Ren, Xiong Zhang, Zheng Li, Jun Miao, Xiaodong Wang

**Affiliations:** 1 Department of Orthopaedics, Affiliated Hospital of Hebei University, No.212, Yuhua Road, Baoding City, 071000 Hebei China; 2 Department of Tuberculosis, Affiliated Hospital of Hebei University, Hebei, China; 3 Department of Rehabilitation, Baoding Third Central Hospital, Tianjin Hospital, No.406, Jiefang Road, Tianjin, 300000 Hebei China; 4 Tianjin Hospital, Tianjin, China

## Abstract

**Objective:**

To study the effect of weight-bearing on lumbar facet joint during lateral bending in sitting position.

**Methods:**

Ten normal healthy people (5 males and 5 females) aged 25–39 years (mean 32 ± 4.29 years) were recruited. CT scanning was used to reconstruct the lumbar spine model, and then dual fluoroscopic image system (DFIS) was used to restore the lumbar facet joint movement in sitting position. Finally, the lumbar facet joint translation distance and rotation angle were measured.

**Results:**

In L3-4 level, the displacement of right facet joint in *Y*-axis was the smallest at 0.05 ± 0.40 mm, the displacement of 0 kg left facet joint in *X*-axis was the largest at 1.68 ± 0.85 mm, and the rotation angle was − 0.57 ± 1.43° to 5.66 ± 2.70° at 10 kg; in L4-5 level, the displacement of right facet joint in *Y*-axis was the smallest at 10 kg, − 0.13 ± 0.91 mm, and the displacement of left facet joint in *Z*-axis was the largest at − 2.11 ± 0.88 mm, and the rotation angle was 0.21 ± 2.14° to 7.89 ± 2.59° at 10 kg; in L5-S1 level, the displacement of right facet joint in *Y*-axis was the smallest at 10 kg, − 0.17 ± 1.10 mm, and the displacement of 0 kg left facet joint in *X*-axis was the largest at 2.19 ± 2.28 mm, and the rotation angle was 0.03 ± 2.02° to 3.98 ± 0.37°.

**Conclusion:**

In sitting position, weight-bearing has certain influence on the displacement of facet joints during lumbar lateral bending movement, and this influence occurs simultaneously in translation and rotation; the left and right facet joints are not symmetrical during lumbar lateral bending movement.

## Background

With the development of society, sitting posture has become the primary position for human learning, living, and working. sitting posture exerts greater pressure on the lumbar spine, making it more susceptible to degenerative changes and subsequently leading to lower back pain [1–5]. Studying the movement patterns of lumbar spine during sitting can further enhance our understanding of the characteristics of lumbar spine motion. The facet joints, as a structures located at the posterior aspect of the lumbar spine, play an important role in load-bearing and maintaining stability. Abnormal movement patterns of the facet joints may lead to joint damage and degeneration, ultimately causing lower back pain. Therefore, investigating the movement patterns of the lumbar facet joints can guide proper movement patterns and also contribute to guiding treatment strategies for related conditions.

Currently, research on the motion patterns of small joints is often limited to 2D methods, such as commonly used X-ray, CT, MRI, etc. However, the motion of lumbar small joints is three-dimensional, not only limited to a specific plane but also involving changes in the entire spatial structure. Therefore, the currently applied research methods are unable to effectively measure the three-dimensional motion of small joints. At the same time, most methods that can be used for 3D motion measurement, such as tracking measurement, often have low accuracy and disadvantages such as invasive operations, making them more suitable for overall spinal motion measurement rather than fine motion structures like small joints. Therefore, we chose the dual fluoroscopic image system that combines 2D and 3D for the measurement of small joints. This method not only has high accuracy but also accurately reproduces the motion of lumbar facet joints in vivo.

In this study, we hypothesize that: 1. The motion pattern of lumbar facet joints will change after loading during sitting posture; 2. There is an asymmetry in the motion pattern of lumbar facet joints between the left and right sides, which exists regardless of loading or non-loading conditions.

## Methods

### Time and place

Ten normal healthy people (5 males and 5 females) aged 25–39 years with an average of 32 ± 4.29 years were recruited for this study. Inclusion criteria: 1. Without heart disease, cerebrovascular disease, liver and kidney disease; 2. BMI between 18.5 and 23.9; 3. *T* value in bone mineral density between − 1 and 1, patients do not have bone metabolic disease. Exclusion criteria: 1. History of lumbar surgery or lumbar trauma; 2. There are spinal diseases, such as idiopathic scoliosis, Humen disease and other diseases that cause lumbar deformities; 3. Pregnancy; 4. Severe osteoporosis and other diseases that may affect the test results.

The study was conducted according to the Declaration of Helsinki (revised 2013). The study was explained to all subjects and informed consent was signed. This study was approved by Tianjin Hospital Tianjin University Research Ethics Committee.

### Reconstruction of 3D model of lumbar vertebra

**Fig. 1:**
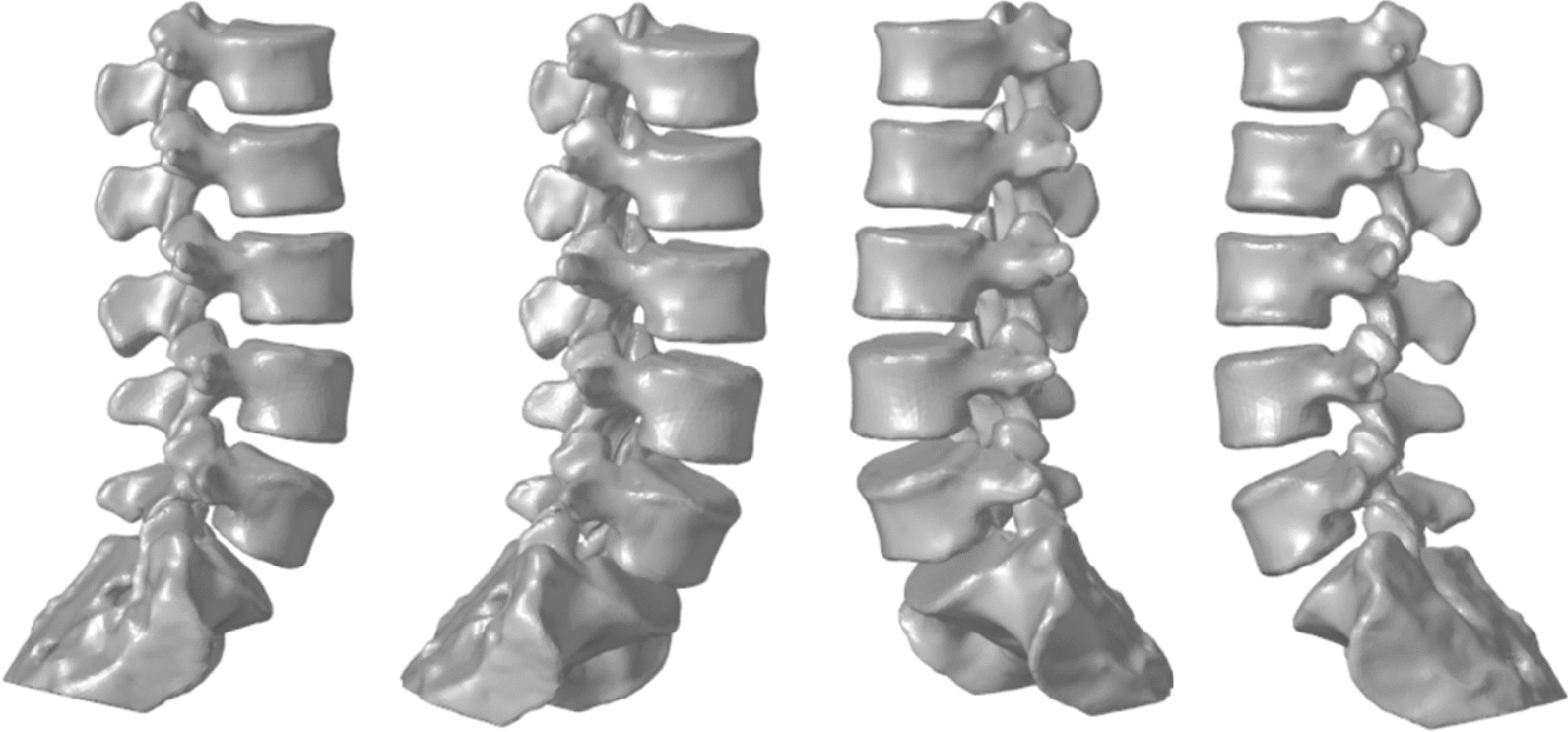
3D model of the spine established by MIMICS software

The subjects completed thin-slice CT scanning in supine position with CT equipment (Sensation16, Siemens, Germany), tube voltage 120 kV, tube current 280 mAs, scanning angle 0°, slice thickness 0.625 mm, resolution 512*512 pixels, window level 50, window width 360. Subjects were scanned in the supine position for 45 s, including the range L1-S1, and the resulting data were stored in DICOM format. The obtained image data were imported into Mimics 21.0 software, and 3D model reconstruction of the lumbar spine was performed by selecting a special bone threshold and extracting the bone model (Fig. 1).

### Establishment of dual fluoroscopic image system (DFIS)

The DFIS consists of two mobile X-ray units: the mobile C-arm X-ray machines used are produced by General Electric Healthcare Group, with both C-arm models being GEOECFluorostar7900. The two C-arms are identical in terms of specifications. The C-arms are designated as F1 and F2 respectively. During the experiment, the planes in which the two C-arms are located are perpendicular to each other, and the line formed by extending the two planes is close to the lumbar vertebrae L3-S1 of the subjects.

**Fig. 2:**
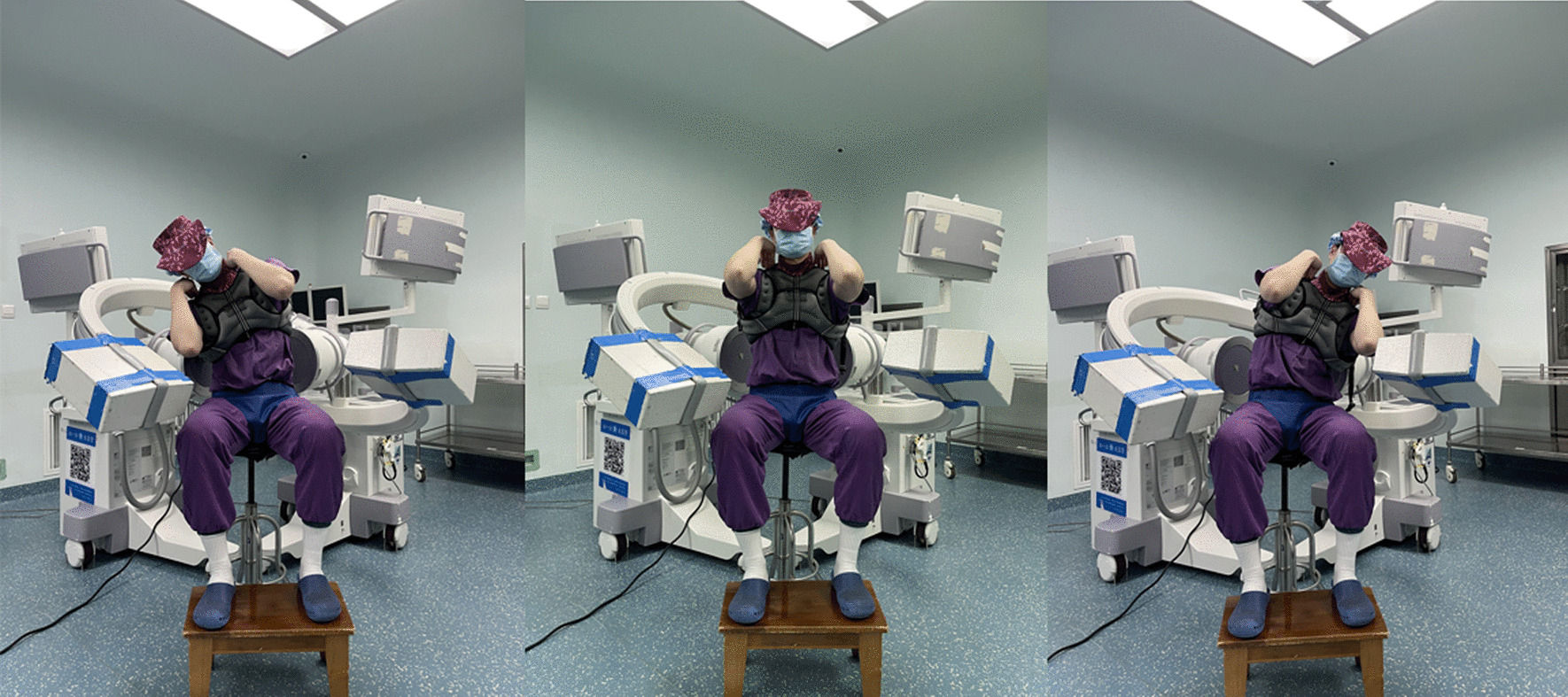
Data collected by subjects in DFIS system consisting of two C-arms

The subjects sit on an adjustable-height chair, adjusted according to their individual height. The subjects' pelvis is fixed, maintaining the position of the thighs parallel to the ground and the lower legs perpendicular to the ground. Both upper limbs are placed on the shoulders to ensure that the lumbar vertebrae are at the center of the collimator field and within the imaging acquisition area. The subjects perform three movements: left lateral bending, neutral position, and right lateral bending, with each movement being held at maximum range. X-ray fluoroscopy (30 frames per second, 8 ms pulse width) was performed at each position for 1 s to obtain clear lumbar X-ray images. The subjects carried a specially designed 10 kg load for 30 min and then underwent X-ray imaging of the same movements again. The image acquisition process was supervised by two spine surgeons to ensure the accuracy of the movements (Fig. 2). The obtained X-ray images were saved in DICOM format and then underwent diffraction removal image processing.

### Establish coordinate system

Rhinoceros 5 (64-bit) software was used to establish a coordinate system. Select the center point of the vertebra to establish a Cartesian coordinate system, with the center point as the origin. Three axis lines are established based on the origin: *X*, *Y*, and *Z*. The *X*-axis (red) is a horizontal line pointing to the left on the coronal plane. The *Y*-axis (green) is a horizontal line pointing backwards on the sagittal plane. The *Z*-axis (blue) is a vertical line pointing towards the head on the sagittal plane. The displacements along each axis are denoted as *x*, *y*, and *z*, respectively. The rotation angles around the *X*, *Y*, and *Z* axes are denoted as *α*, *β*, and *γ*, respectively. Positive displacements are in the same direction as the arrow, while negative displacements are in the opposite direction. Clockwise rotation is denoted as positive, while counterclockwise rotation is denoted as negative. Displacements are noted in millimeters (mm), while rotation are noted in degrees (°). Copy the established coordinate system with the origin located at the midpoint between the articular processes below the cranial vertebral body and above the caudal vertebral body. At this point, the coordinate system for the articular process joint is established, which can be used to measure the displacement distance and rotational angle of the articular process joint.

### Reproduction of lumbar facet joint kinematics in different seated positions:

**Fig. 3:**
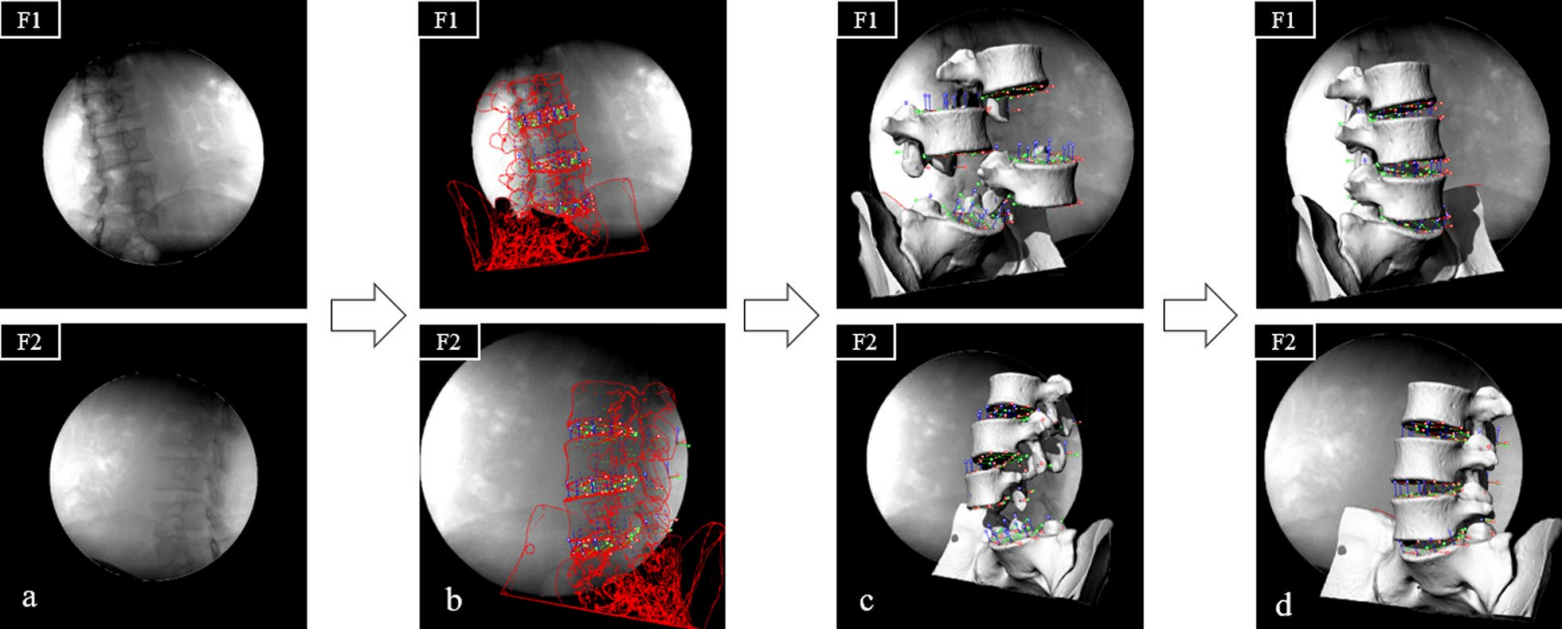
Schematic diagram of the process of matching the lumbar spine model of 2D-3D through the software. a. import the X-rays acquired by the two C-arms of F1 and F2 into Rhinoceros 5, b. draw the boundary of the lumbar spine in the software, c. import the 3D model reconstructed in Mimics software into Rhinoceros 5, d. adjust the 3D model in the F1 and F2 perspectives until the vertebral body and 3D model in the background overlap. At this point, the movement distance and rotation angle of lumbar facet joints can be measured in the software

Import the X-ray images with diffraction removed into Rhinoceros 5 (64-bit) and simulate the X-ray emission and receiving devices in the software according to the method proposed by LI et al. [6]. The X-ray images serve as background images in the software, and the anatomical contours of the vertebral body, facet joints, spinous processes, etc. are delineated using the relevant tools in the software, completing the 2D modeling of the lumbar spine model. Import the CT 3D model into the modeling software. Adjust the position of each vertebral body based on the anatomical structure of the lumbar spine, ensuring complete overlap with the anatomical contours of the background image, thereby achieving the 2D-3D matching of vertebral body positions during different movements. The restored lumbar spine motion states in different positions (left bending, neutral position, right bending) can be obtained (Fig. 3).

### Measurement of three-dimensional lumbar spine model data

By measuring the relative position changes of the lumbar zygapophyseal joints, data on the corresponding motion changes can be obtained. Specifically, the position comparison is made between the L4 superior articular process and the L3 inferior articular process, the L5 superior articular process and the L4 inferior articular process, and the S1 superior articular process and the L5 inferior articular process. The motion characteristics of the lumbar zygapophyseal joints during sitting position are studied by comparing the data of left bending-right bending without load and left bending-right bending with a load of 10 kg.

### Data statistics and analysis

Statistical analysis was performed using SPSS 26.0 (IBM, Armonk, NY, USA). The dependent variable was the movement change, while the independent variables were the load and vertebral level. The data were found to follow a normal distribution based on the Kolmogorov–Smirnov test. The paired *t*-test was used to compare the displacement and rotational angles of the facet joints at L3/4, L4/5, and L5/S1 under 0 kg and 10 kg load conditions. A significance level of *P* < 0.05 was considered statistically significant, and continuous variables were expressed as $X\overline{x }$±S.

## Results

### Left–right bending movements at the L3-4 segment

When there is no load, the displacement on the left side along the coronal axis (*X*-axis), sagittal axis (*Y*-axis), and vertical axis (*Z*-axis) is 1.68 ± 0.85 mm, − 0.78 ± 1.10 mm, and − 1.34 ± 2.84 mm, respectively.The displacement on the right side along the coronal axis (*X*-axis), sagittal axis (*Y*-axis), and vertical axis (*Z*-axis) is 1.45 ± 0.88 mm, 0.52 ± 0.47 mm, and 0.49 ± 2.07 mm, respectively. The rotation degrees along the *α*, *β*, and *γ* axes on both sides are − 1.41 ± 3.55°, 5.66 ± 2.70°, and − 2.56 ± 2.05°, respectively. When there is a load of 10 kg, the displacement on the left side along the coronal axis (*X*-axis), sagittal axis (*Y*-axis), and vertical axis (*Z*-axis) is 0.40 ± 0.74 mm, − 0.30 ± 0.65 mm, and − 0.97 ± 1.02 mm, respectively. The displacement on the right side along the coronal axis (*X*-axis), sagittal axis (*Y*-axis), and vertical axis (*Z*-axis) is 0.69 ± 0.53 mm, 0.05 ± 0.40 mm, and 0.05 ± 1.75 mm, respectively. The rotation degrees along the *α*, *β*, and *γ* axes are − 1.01 ± 2.64°, 2.22 ± 3.70°, and − 0.57 ± 1.43°, respectively.

### Left–right bending movements at the L4-5 segment

When there is no load, the displacement on the left side along the coronal axis (*X*-axis), sagittal axis (*Y*-axis), and vertical axis (*Z*-axis) is 0.91 ± 1.39 mm,  − 0.35 ± 1.77 mm, and − 2.11 ± 0.88 mm, respectively. The displacement on the right side along the coronal axis (*X*-axis), sagittal axis (*Y*-axis), and vertical axis (*Z*-axis) is 0.62 ± 1.14 mm, 0.53 ± 0.87 mm, and 1.61 ± 1.00 mm, respectively. The rotation degrees along the *α*, *β*, and *γ* axes on both sides are − 0.39 ± 1.52°, 7.89 ± 2.59°, and − 0.84 ± 3.65°, respectively. When there is a load of 10 kg, the displacement on the left side along the coronal axis (*X*-axis), sagittal axis (*Y*-axis), and vertical axis (*Z*-axis) is 0.74 ± 1.67 mm, 0.28 ± 0.53 mm, and − 1.72 ± 2.30 mm, respectively. The displacement on the right side along the coronal axis (*X*-axis), sagittal axis (*Y*-axis), and vertical axis (*Z*-axis) is − 0.36 ± 1.09 mm, − 0.13 ± 0.91 mm, and 1.17 ± 1.02 mm, respectively. The rotation degrees along the *α*, *β*, and *γ* axes are − 1.25 ± 2.58°, 3.67 ± 5.79°, and 0.21 ± 2.14°, respectively.

### Left–right bending movements at the L5-S1 segment

**Fig. 4:**
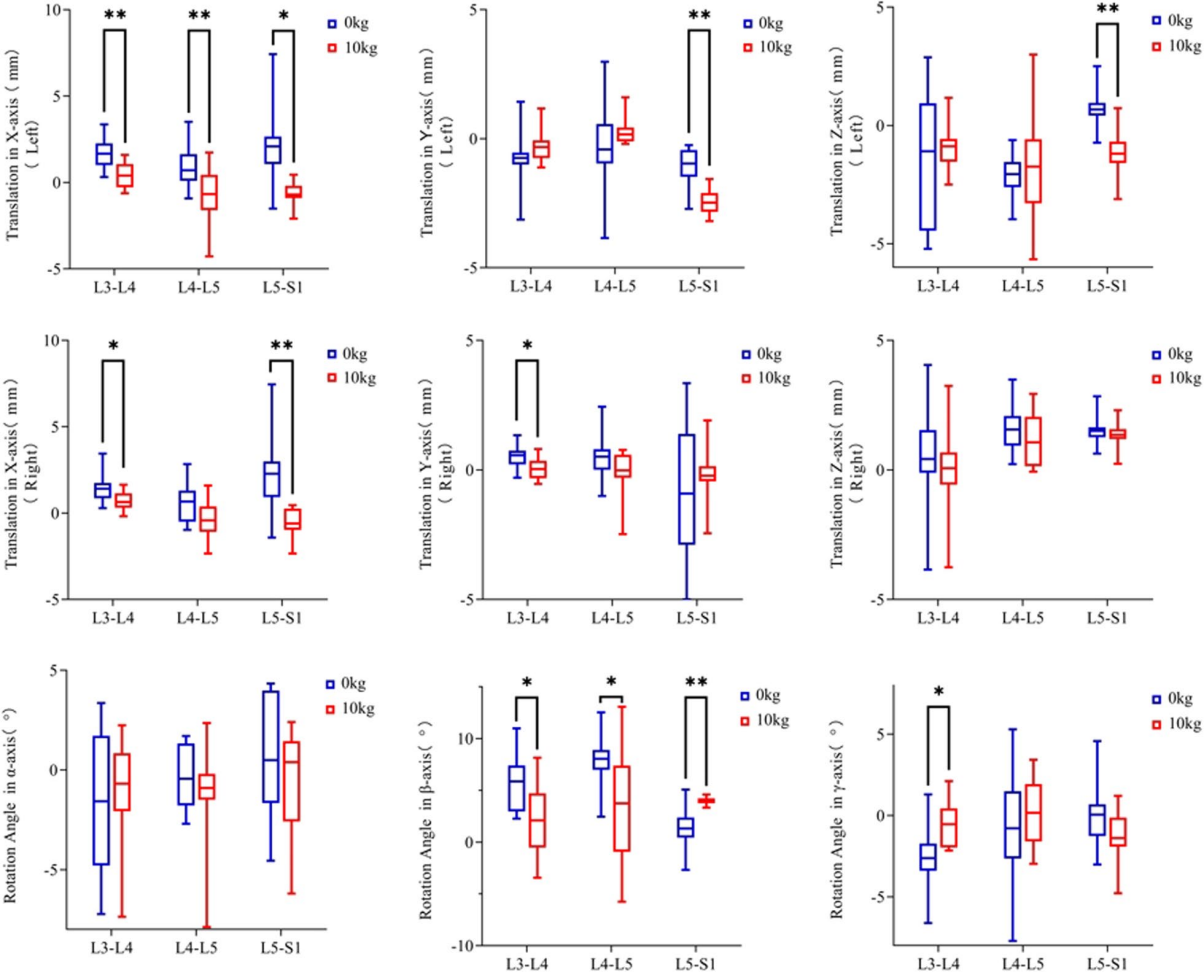
Translation distance and rotation angle of left and right facet joints at different lumbar levels

When there is no load, the displacement on the left side along the coronal axis (*X*-axis), sagittal axis (*Y*-axis), and vertical axis (*Z*-axis) is 2.19 ± 2.28 mm, − 1.07 ± 0.79 mm, and 0.72 ± 0.81 mm, respectively. The displacement on the right side along the coronal axis (*X*-axis), sagittal axis (*Y*-axis), and vertical axis (*Z*-axis) is 2.35 ± 2.37 mm, − 0.86 ± 2.88 mm, and 1.53 ± 0.57 mm, respectively. The rotation degrees along the *α*, *β*, and *γ* axes on both sides are 0.60 ± 2.95°, 1.28 ± 2.07° and 0.03 ± 2.02°, respectively. When there is a load of 10 kg, the displacement on the left side along the coronal axis (*X*-axis), sagittal axis (*Y*-axis), and vertical axis (*Z*-axis) is − 0.65 ± 0.69 mm, − 2.45 ± 0.48 mm, and − 1.14 ± 1.03 mm, respectively. The displacement on the right side along the coronal axis (*X*-axis), sagittal axis (*Y*-axis), and vertical axis (*Z*-axis) is 0.57 ± 0.85 mm, − 0.17 ± 1.10 mm, and 1.35 ± 0.51 mm, respectively. The rotation degrees along the *α*, *β*, and *γ* axes are − 0.56 ± 2.71°, 3.98 ± 0.37°, and − 1.35 ± 1.62°, respectively. (Table 1, Fig. 4).

**Table 1:** Displacement and rotation of left and right facet joints at different lumbar segments

Segment	L3-4			L4-5			L5-S1		
Loading	0 kg	10 kg	P	0 kg	10 kg	P	0 kg	10 kg	P
Translation									
Left (mm)									
X	1.68 ± 0.86	0.40 ± 0.74	< 0.01	0.91 ± 1.37	− 0.74 ± 1.67	0.28	2.19 ± 2.28	− 0.65 ± 0.69	< 0.01
*Y*	− 0.78 ± 1.10	− 0.30 ± 0.65	0.25	− 0.35 ± 1.77	0.28 ± 0.53	0.30	− 1.07 ± 0.79	− 2.45 ± 0.49	< 0.01
*Z*	− 1.34 ± 2.84	− 0.97 ± 1.02	0.70	− 2.12 ± 0.88	− 1.72 ± 2.30	0.62	0.72 ± 0.81	− 1.14 ± 1.04	< 0.01
Translation									
Right (mm)									
*X*	1.45 ± 0.89	0.69 ± 0.53	0.03	0.62 ± 1.14	− 0.36 ± 1.09	0.06	2.36 ± 2.37	− 0.57 ± 0.85	< 0.01
*Y*	0.52 ± 0.47	0.05 ± 0.40	0.03	0.53 ± 0.87	− 0.13 ± 0.91	0.11	− 0.86 ± 2.88	− 0.17 ± 1.10	0.49
*Z*	0.49 ± 2.07	0.05 ± 1.75	0.01	1.61 ± 1.00	1.17 ± 1.02	0.35	1.53 ± 0.57	1.35 ± 0.51	0.47
Rotation Angle (°)									
*α*	− 1.41 ± 3.55	− 1.01 ± 2.64	0.78	− 0.39 ± 1.52	− 1.25 ± 2.59	0.37	0.60 ± 2.95	− 0.56 ± 2.71	0.37
*β*	5.67 ± 2.70	2.22 ± 3.70	0.03	7.89 ± 2.59	3.67 ± 5.79	0.05	1.28 ± 2.08	3.98 ± 0.37	< 0.01
*γ*	− 2.65 ± 2.05	− 0.57 ± 1.43	0.02	− 0.85 ± 3.65	0.21 ± 2.14	0.44	0.03 ± 2.02	− 1.35 ± 1.62	0.11

## Discussion

Low back pain is now one of the most common symptoms at work. According to the Bureau of Labor Statistics, musculoskeletal disorders (MSD) accounted for approximately 31% of all work-related injuries in the United States in 2015, with low back-related injuries comprising 40% of all MSDs. Low back pain imposes significant physiological and psychological burdens on patients, as well as substantial social impacts. A research survey conducted in the US revealed that the annual cumulative cost of low back pain exceeds $100 billion, two-thirds of which are indirect costs resulting from wage loss and decreased productivity [7]. The lumbar facet joints are one of the important factors contributing to low back pain [8–11], often referred to as "facet joint syndrome." Studies suggest that approximately 16–40% of chronic low back pain is caused by facet joints [12–14]. Research on the movement characteristics of the lumbar facet joints can help us better understand their motion during daily activities, aiding in the analysis of the degenerative causes of facet joints and providing a theoretical basis for the prevention and treatment of facet joint disorders.

Current research often focuses on the non-weight-bearing movement variations of the lumbar facet joints during standing, while there is a lack of relevant studies on the movement patterns of the lumbar facet joints under weight-bearing conditions during sitting. Investigating the movement patterns of the facet joints under weight-bearing conditions during sitting would contribute to a comprehensive understanding of lumbar spine motion and provide better guidance for proper movement patterns and rehabilitation treatments. At present, most studies are limited by conditions, often in two-dimensional plane or in vitro, can not effectively reduce the movement of lumbar joints in vivo, have lower accuracy. Our study utilized the DFIS method to restore the motion changes of the lumbar facet joints in vivo. This method has a high level of accuracy, with reported displacement errors of 0.3 mm and rotational errors of 0.7° [6, 15], enabling a more accurate reflection of the motion changes of the lumbar facet joints in vivo.

Chowdhury et al. observed the changes in the facet joints movements during weight-bearing exercise in 11 healthy subjects [16]. The study found that small joints had significant load effects in lateral bending and superior-inferior translation. After weight-bearing, the L5-S1 joint had greater lateral bending and twisting than the L2-L3, L3-L4, and L4-L5 joints. The authors suggested that the kinematics of lumbar spine small joints were influenced by the magnitude of the load and the direction of adjacent joints. As the orientation of the L5-S1 small joints was more coronal than the L2-L3, L3-L4, and L4-L5 joints, greater lateral bending and twisting were produced. Our study also showed that weight-bearing had a significant effect on facet joint movements, except for the *Y* and *Z* axes in the left L5-S1 segment, where the displacement of small joints was significantly reduced at 10 kg compared to 0 kg. This may be due to the increased pressure on small joints and the decreased gap between the upper and lower facets caused by weight-bearing, resulting in reduced small joint displacement. This effect was more pronounced in the L3-4 and L4-5 segments, which differed from Chowdhury et al.'s finding that weight-bearing had a more significant effect on the L5-S1 segment. We believe that this difference may be due to the lack of fixation of the pelvis and restriction of hip joint movement in Chowdhury et al.'s study, which may have affected the motion of the L5-S1 facet joints; Additionally, the weight used in Chowdhury et al.'s study was different from that in our study, and we speculate that a higher weight may be more likely to induce small joint motion changes. Chowdhury et al.'s study did not measure the rotation angle of small joints, but we found that the rotation angles of small joints in lateral bending were not consistent. In the L3-4 and L4-5 segments, the rotation angle decreased with increasing weight, but in the L5-S1 segment, weight-bearing increased the rotation angle. We believe that this is due to the unique anatomical structure of the L5-S1 joint, which is affected by the sacroiliac joint and changes the motion pattern of the L5-S1 small joints, leading to an increase in rotation angle after weight-bearing.

Song et al. measured the movement of the lumbar facet joints during flexion and extension of the spine in the standing position [17]. After comparing the measurements with 0 kg, 5 kg, and 10 kg loads, the researchers concluded that the load did not increase the range of motion of the lumbar facet joints. This may be due to compensatory effects from muscles and ligaments. Wen et al. studied the effects of loads of 0 kg, 5 kg, and 10 kg on the small joints of the spine during standing posture and concluded that the loads have an impact on the coupled motion of the spine, but the effect on the small joints during spinal lateral bending is not significant [18]. In this study, we believe that the movement of facet joints is influenced by load when sitting. When the load is 10 kg, the displacement of the facet joint was significantly reduced compared to 0 kg. This result is observed in the *X*-axis of the small joints on the left side of the L3/4 segment, the right side of the L3/4 segment, the *Y*-axis of the small joints on both sides of the L3/4 segment, the *Z*-axis of the small joints on both sides of the L3/4 segment, the *X*-axis of the small joints on the left side of the L5/S1 segment, the *Y*-axis of the small joints on both sides of the L5/S1 segment, the *Z*-axis of the small joints on both sides of the L5/S1 segment, and the *X*-axis of the small joints on the right side of the L5/S1 segment. This is different from previous research results. This is different from previous research findings. We believe that the reason for this difference lies in the different postures. When sitting, the pressure on the lumbar spine is greater than when standing, and the range of motion of the lumbar facet joints is relatively larger compared to when standing [19, 20]. Therefore, the effect of load on the lumbar facet joints may be greater during sitting. In addition, the tilt of the pelvis also contributes to the differences in results. When subjects are in a standing position, the pelvis cannot be completely fixed, and spinal lateral bending often accompanies partial pelvic tilt. However, when sitting, the pelvis is fixed in a specific seat and cannot tilt, resulting in less influence on spinal lateral bending. In our study, we found that when a load of 10 kg was applied, the lumbar facet joints exhibited a pattern of lateral translation accompanied by rotation. This lateral bending pattern with translation and rotation in the lumbar facet joints is similar to that observed without load. Among the lumbar facet joints, more parallel displacement occurred at the L3-4 and L4-L5 levels, which may be due to the limited mobility of the L5/S1 facet joints caused by pelvic fixation, resulting in relatively less motion at the L5-S1 level compared to the levels above.

Many studies have found asymmetry in the small joints on the left and right sides [21–23]. Leng et al. suggested that the asymmetry of the lumbar facet joints is correlated with lumbar spondylolisthesis [23]. Wang et al. found a correlation between the asymmetry of the lumbar facet joints and spinal canal stenosis [24]. Kou et al. investigated the asymmetry of lumbar facet joint motion during standing and found that the most pronounced asymmetry occurred in the L3-L4 and L5-S1 facet joints [25]. In our study, we also observed an asymmetrical movement of the lumbar zygapophyseal joints during lateral flexion in the seated position. In the segments of L3-4, L4-5, and L5-S1 without load, both sides of the lumbar zygapophyseal joints exhibited asymmetrical movement patterns. This phenomenon persisted even when a load of 10 kg was added. The load did not affect the asymmetrical movement of the lumbar facet joints. Previous studies have shown that there is anatomical asymmetry in the lumbar zygapophyseal joints [26]. We believe that this anatomical asymmetry in the facet joints causes the asymmetrical movement patterns. This anatomical asymmetry does not change with the presence of a load, and therefore, the load does not affect the asymmetrical movement of the facet joints.

There are several limitations in this study. Firstly, the sample size is small. Due to the complexity of the experiment, the sample size in this study was only 10, which may have led to insignificant results in some cases. Future research can increase the sample size for analysis. Secondly, we only measured the lateral bending motion of the lumbar spine in a seated position. In future studies, we can include rotational and other daily movements for correlation analysis. Lastly, due to the limitations of the projection range, this study only measured the facet joints of the lumbar spine at L3-S1. Future research can measure the facet joints of the upper lumbar spine to better demonstrate the overall motion pattern of the lumbar spine. Despite these limitations, this study, for the first time, investigated the influence of load on lateral bending motion of the lumbar facet joints in a seated position through non-invasive methods. This has important implications for the prevention of lumbar facet degeneration and the treatment of related diseases.

## Conclusion

1. When sitting, load-bearing has a certain impact on the displacement of the facet joints during lumbar lateral bending, and this impact occurs simultaneously in translation and rotation.
2. The facet joints on the left and right sides are not symmetrical during lumbar lateral bending.

## Data Availability

The datasets used and/or analysed during the current study available from the corresponding author on reasonable request.
